# Adherence to and impact of home-based high-intensity IMT in people with spinal cord injury: a pilot study

**DOI:** 10.1038/s41394-022-00551-5

**Published:** 2022-10-30

**Authors:** Anne E. Palermo, Mark S. Nash, Neva J. Kirk-Sanchez, Lawrence P. Cahalin

**Affiliations:** 1 Neuroscience Research Australia, Sydney, NSW Australia; 2 University of New South Wales, Sydney, NSW Australia; 3 University of Miami Miller School of Medicine, Department of Physical Therapy and The Miami Project to Cure Paralysis, Miami, FL USA; 4 University of Miami Miller School of Medicine, Department of Physical Therapy, Miami, FL USA

**Keywords:** Rehabilitation, Preventive medicine

## Abstract

**Study design:**

The pilot study was completed in 5 phases (Control and 4 phases of IMT) incorporating assessments at Baseline 1 (BL1), BL2, Follow-up 1 (F1), F2, F3, and F4.

**Objective:**

To assess the adherence and impact of a daily high-intensity (80% of max) inspiratory muscle training (IMT) home program with once weekly supervision for people with spinal cord injury (SCI).

**Setting:**

Assessments: research institution or zoom. IMT: participant’s home.

**Methods:**

Participants completed daily IMT in IMT Phase 1 and 2, once weekly in IMT Phase 3, self-selected frequency in IMT Phase 4. All phases had one weekly supervised session except IMT Phase 4. Primary outcomes included adherence and a difficulty score [DS (0- not difficult to 10- the most difficult)]. Secondary outcomes included respiratory function and seated balance.

**Results:**

Data from 10 people with chronic SCI (>1 year) (Cervical level of injury: 6, AIS: A-B, injury duration: 10.9 years 95% CI [3.9, 18.1]) were used in the analysis. Participants completed 69% of their training days in IMT Phase 1 and 65% overall reporting an average DS of 7.4 ± 1.4. Only one participant completed training during IMT Phase 4. One participant’s training load was reduced due to suspected overtraining. Maximal inspiratory pressure (MIP), sustained MIP (SMIP), and total power (TP), improved significantly (*p* < 0.05) from BL2 to F1.

**Conclusion:**

Our data suggest that people with SCI can perform high-intensity IMT at home to improve inspiratory performance. It is strongly recommended that participants be intermittently monitored for adherence and safety.

ClinicalTrials.gov Registration number: NCT04210063.

## Introduction

**Fig. 1:**
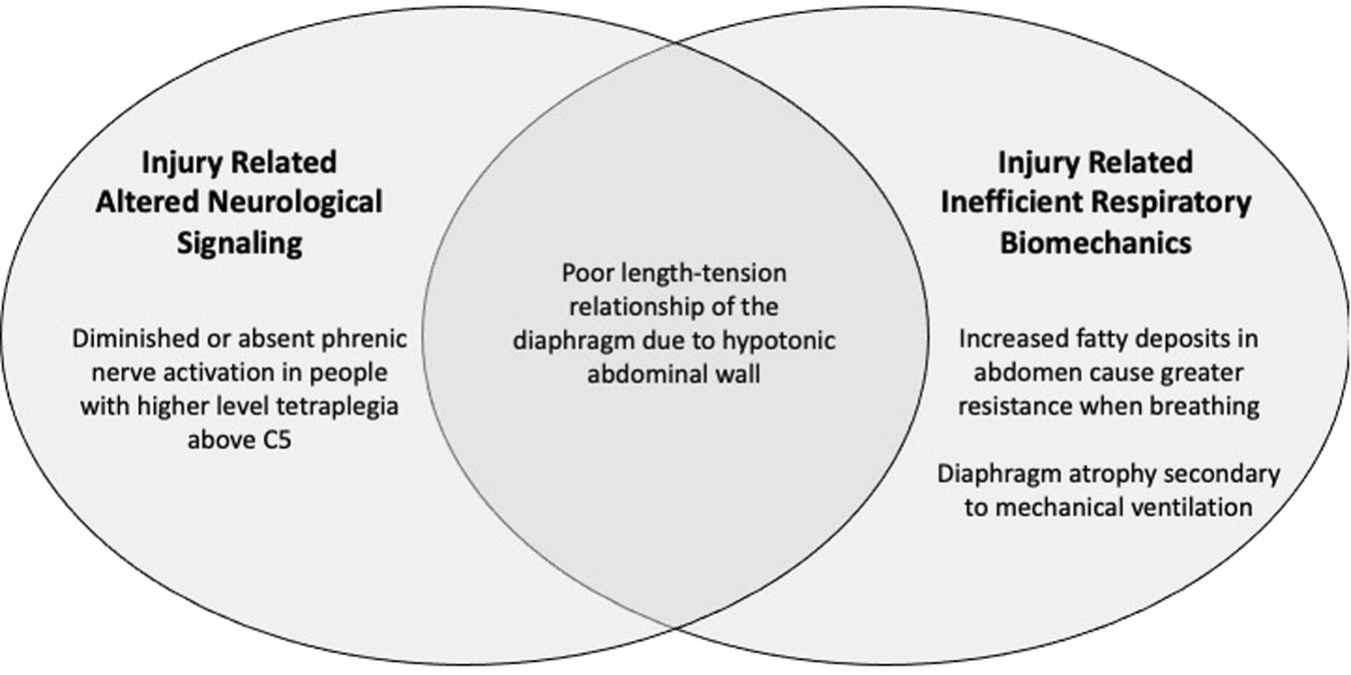
Altered respiratory function. This figure summarizes the impact of SCI on RF described in a previous publication [4].

People with spinal cord injury (SCI) sustain weakened respiratory musculature due to altered neurological and biomechanical functions (Fig. 1) [1–4]. Respiratory complications (RC) have been a leading cause of morbidity and mortality for individuals with SCI for more than 40 years [5–8]. Further, individuals with SCI are less likely to receive general preventative care than neurologically intact individuals [9]. Insurance, lack of funds, physical entry to buildings or rooms, and transportation act as barriers to healthcare access for this population [9–12].

Inspiratory muscle training (IMT) performed alone or with expiratory muscle training (EMT), termed respiratory muscle training (RMT), may be an effective low-cost intervention with potential to circumvent healthcare access issues for individuals with SCI. Studies show that RMT improves pulmonary function (PF) and respiratory muscle strength while decreasing the risk of RC [13–16]. Continued high rates of RC and mortality suggest that widespread implementation of RMT has not occurred despite growing evidence of benefits for individuals post-SCI [9–11, 17].

The benefits of IMT may not be limited to respiratory function (RF). The diaphragm plays a dual role in respiration and balance [18]. Inspiratory muscle weakness has been linked to poor balance in people with and without SCI [19–23]. Relationships among functional seated balance (FSB) and the quality of life of people with SCI have also been reported [18–21, 23–30]. The impact of IMT on FSB and quality of life for individuals with SCI has not been investigated.

A home-based RMT program, targeting RF and balance, may bypass obstacles encountered when people with SCI seek care. However, the program adherence of a person with SCI completing unsupervised RMT is not well documented [31–35]. Recent studies implementing unsupervised RMT in people with SCI only intermittently reported RF outcomes and none mentioned balance [35], warranting a more thorough investigation of the impact of unsupervised IMT on RF and FSB.

The primary purpose of this study was to investigate the adherence to daily home-based, high-intensity IMT completed by individuals with chronic SCI. We hypothesized that individuals would be 70% compliant with the daily training program, allowing for two missed sessions per week without compromising the five-day threshold found in successful trials [15, 31]. We also hypothesized that IMT would positively impact RF and seated balance compared to a control period.

## Methods

The protocol was approved by a university ethics board and registered on ClinicalTrials.gov. Individuals were recruited for the study from a volunteer database maintained at the research institution. Volunteers recruited from an institutional database over 18 years old with any level of injury (LOI) and impairment grades A-C based on the American Spinal Injury Association Impairment Scale participated in this study. Individuals who required mechanical ventilation, were taking beta-blockers, who had a pacemaker, or who were being treated for an acute pressure sore, RC, or urinary tract infection were excluded from the study. Additionally, individuals who were able to stand did not qualify. See Appendix 1 for more information.

The primary outcomes of the study were the percent of completed sessions and the difficulty score (DS). The DS was reported by the participant in the training log after each session by circling a number along a 0 (not difficult at all) to 10 (the most difficult) numbered scale, like a visual analog scale. Session information was obtained from the training logs of participants and confirmed through the PrO2 (PrO2FIT Inc, Smithfield, RI) cloud-based system.

The secondary outcomes in this study were respiratory pressures and volumes and FSB [1, 2]. Inspiratory measures obtained through the PrO2 device included MIP (cmH2O), sustained MIP (SMIP) (PTU), inspiratory duration (ID)(s), and Total Power (TP) (PTU). MIP, the greatest pressure generated in the first two seconds of an inspiratory maneuver, reflected inspiratory muscle strength [36, 37]. SMIP, the pressure generated from residual volume (RV) to total lung capacity (TLC), reflected the ability of inspiratory muscles to maintain pressure over time [38–40]. ID measures the time of maximal inspiratory effort [38–40]. TP is the summation of SMIPs throughout a training session. The PF outcomes included Maximal Expiratory Pressure (MEP) (cmH2O) recorded on the Micro RPM device (Vyaire Medical, Mettawa, IL), and forced vital capacity (FVC) (L), forced expiratory volume in one second (FEV1) (L), and peak expiratory flow (PEF) (L/sec) assessed via the Micro I (Vyaire Medical, Mettawa, IL).

All respiratory testing was performed with participants seated in their personal wheelchairs. For inspiratory testing, participants were instructed to exhale to RV before inhaling as hard and for as long as possible to TLC. For MEP, participants were instructed to inhale to TLC and exhale maximally for several seconds. For expiratory volume testing, participants were asked to inhale to TLC before exhaling as hard and for as long as possible to RV. At least three but no more than seven testing breaths were performed until consecutive values differed by <10%. The highest values from a single breath were used for analysis.

The Function in Sitting Test for individuals with SCI (FIST-SCI) quantified FSB [41]. The FIST-SCI is a SCI-specific version of the FIST [42–45]. This 14-item outcome measure is scored on a scale from 0 to 4, with a total possible score of 56 (higher scores indicate greater balance) [41]. The FIST-SCI has excellent reliability and moderate validity [41].

**Fig. 2:**
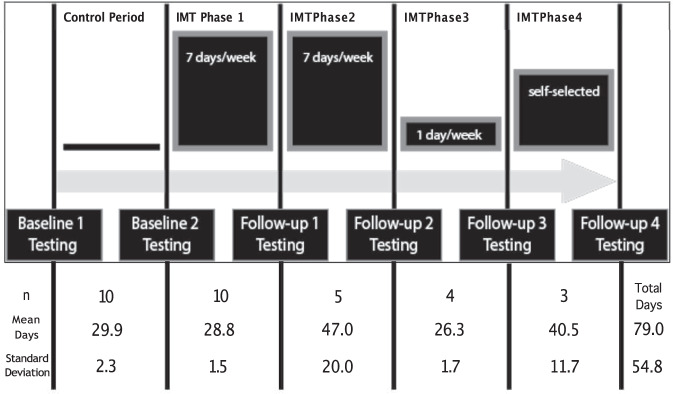
Protocol design. Training frequencies for each IMT Phase are represented in the training blocks along with the number of participants who performed IMT during each phase (n) and the average number of days in each phase.

The study, initiated in December 2019, followed a repeated measures design assessing inspiratory muscle performance, PF, and FSB at multiple time points. Alterations to the initial protocol are detailed in Appendix 1. Ultimately, the study consisted of one control phase and 4 IMT phases separated by 6 testing days (baseline 1,2: BL1, BL2; follow-up 1-4: F1-F4) (Fig. 2). In-person follow-up testing included assessment of all measures, while virtual follow-up sessions held due to COVID-19 included remote assessment of inspiratory muscle performance.

Participants used the PrO2FIT, a flow-resisted inspiratory device with a 2mm-leak, for training. Data from the device was transmitted to the PrO2FIT application on a Bluetooth-paired tablet providing live visual and auditory feedback for each breath. Inspiratory data was also stored in the cloud for subsequent interrogation. IMT training sessions throughout the trial were performed in the home environment and consisted of 42 breaths, completed in 7 sets of 6 breaths, at 80% of the subjects’ maximal baseline effort for the day. Rest time decreased after each set, starting at 40 s and ending with 5 s. The protocol, modeled after one used by Formiga et al. [46], had a similar number of target breaths per week as a successful SCI clinical trial [15].

The a priori study plan included reporting the percentage of completed sessions compared to target sessions and the DS, and analysis of differences in RF and FSB with repeated measures ANOVA of 21 participants. However, the limited sample due to COVID-19 and variable data captured at modified testing time points would not allow for well powered analyses. As such, only outcomes with 10 participants were analyzed by repeated measures ANOVA (MIP, SMIP, ID) for BL1, BL2, and F1, or paired T-Test (TP) between BL2 and F1. Bonferroni post-hoc testing assessed differences between time points if a significant effect was found. Percent of predicted (POP) values were calculated for SMIP [47], MIP [48], MEP [48], FVC [49], FEV1 [49], and PEF [49]. SMIP is the only variable lacking a SCI-based predictive equation, thus an equation created for neurologically intact individuals was used [47]. Post-hoc observed power analyses calculated for the ANOVA analyses completed with SPSS (Version 26) were >0.80.

## Results

**Fig. 3:**
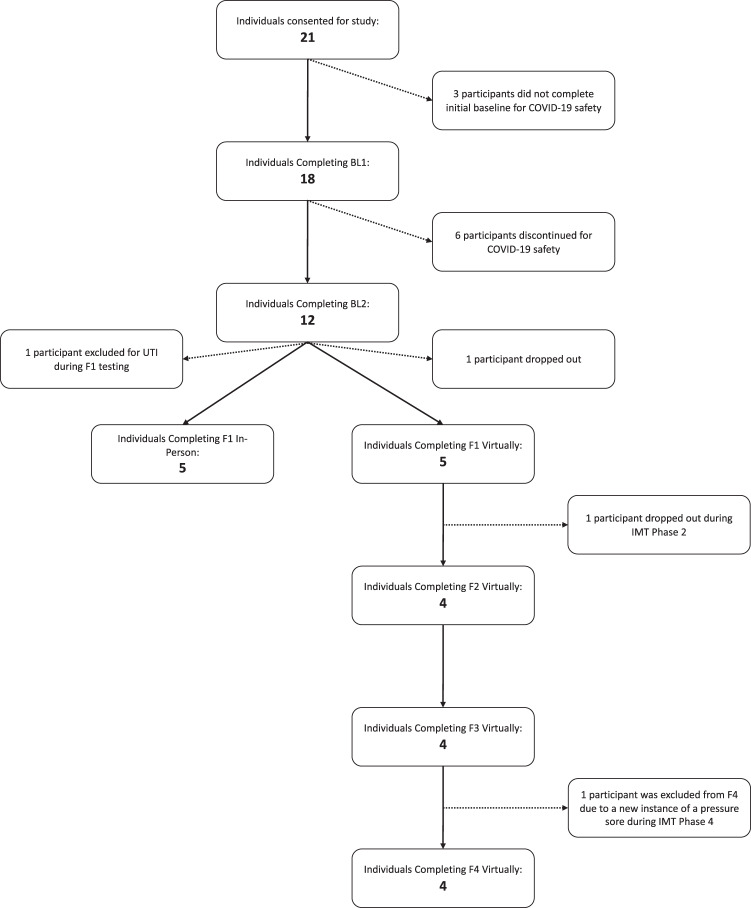
Protocol flow diagram. One participant had an acute urinary tract infection [35] at Follow-up 1 (F1) and his data were not included in the analyses. Both participants who dropped out of the study, one during IMT Phase 1 and the other during IMT Phase 2, cited personal reasons relating to the COVID-19 pandemic. Another participant experienced an acute pressure sore during IMT Phase 4 and their data were not included beyond F3.

Ten of the 12 participants enrolled in IMT Phase 1 completed at least the inspiratory assessments of F1 (Fig. 3). Data from those 10 individuals were included in the analyses. Five participants completed one month of IMT and five participants completed more than one month of training. Descriptive characteristics of the sample can be found in Table 1. Three participants completed all study phases and follow-up testing.

**Table 1:** Sample descriptive characteristics.

	Total sample		1-month of IMT		>1-month of IMT	
	count/mean	95% CI	count/mean	95% CI	count/mean	95% CI
*N*	10		5		5	
Cervical LOI	6		1		5	
Female	2		1		1	
Current smoke/vape	2		1		1	
Age	34.0	[30.0, 38.0]	32.6	[24.9, 40.3]	35.4	[29.0, 41.8]
Duration of Injury (yrs)	10.9	[3.9, 18.1]	12.6	[−2.9, 28.1]	9.2	[−0.9, 19.3]
Height (cm)	172.5	[163.9, 181.2]	174.1	[156.0, 192.2]	171	[158.2, 184.0]
Mass (Kg)	62.9	[55.4, 70.4]	60.3	[50.0, 80.5]	60.5	[49.4, 71.7]

*LOI* level of injury.

Participants completed 69.3% of the target sessions in the first month of daily IMT, which equated to an average training frequency of 4.9 days per week. Training adherence for all stages of training was 65.6%. The average DS per session was 7.4 ± 1.4, however, participants only recorded a DS for 73.3% of their completed sessions.

One participant (P6- represented in green in the figures) with an initial 2-week compliance of 100% and a history of smoking (>20 years) demonstrated worsening RF in the first two weeks. He was instructed to train every other day, which he followed with 100% compliance. Ultimately, the TP, SMIP, ID, FEV1, and FIST-SCI values of P6 increased from BL2 to F1 while, MIP, FVC, and MEP decreased. The PEF for P6 was 0.2% less at F1 than BL2.

**Fig. 4:**
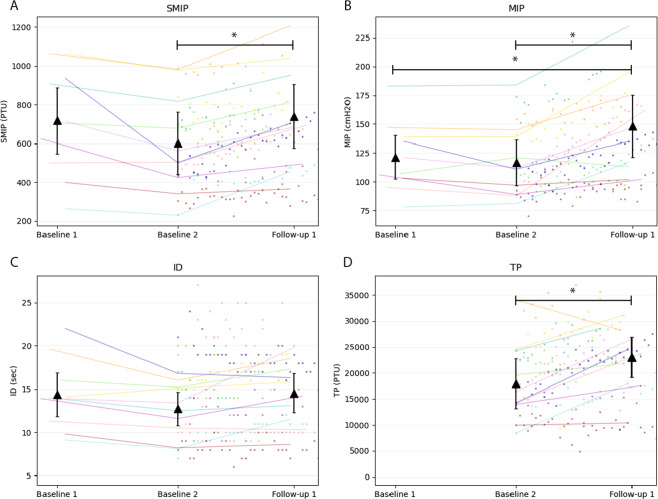
Change of inspiratory outcomes (*n* = 10). Graphical depiction of inspiratory outcomes from Baseline 1 to Follow-up 1 of: (**A**) Sustained Maximal Inspiratory Pressure (SMIP), (**B**) Maximal Inspiratory Pressure (MIP), (**C**) Inspiratory Duration (ID), and (**D**) Total Power (TP). Each color represents data from a single participant with lines showing change between assessment values and dots representing the highest value of the variable achieved per session. The mean and 95% confidence intervals are plotted for each assessment day as black triangles. Participant 6 is represented by the color green. The * shows a significant difference in post-hoc analyses (*p* < .05). PTU pressure time units.

**Fig. 5:**
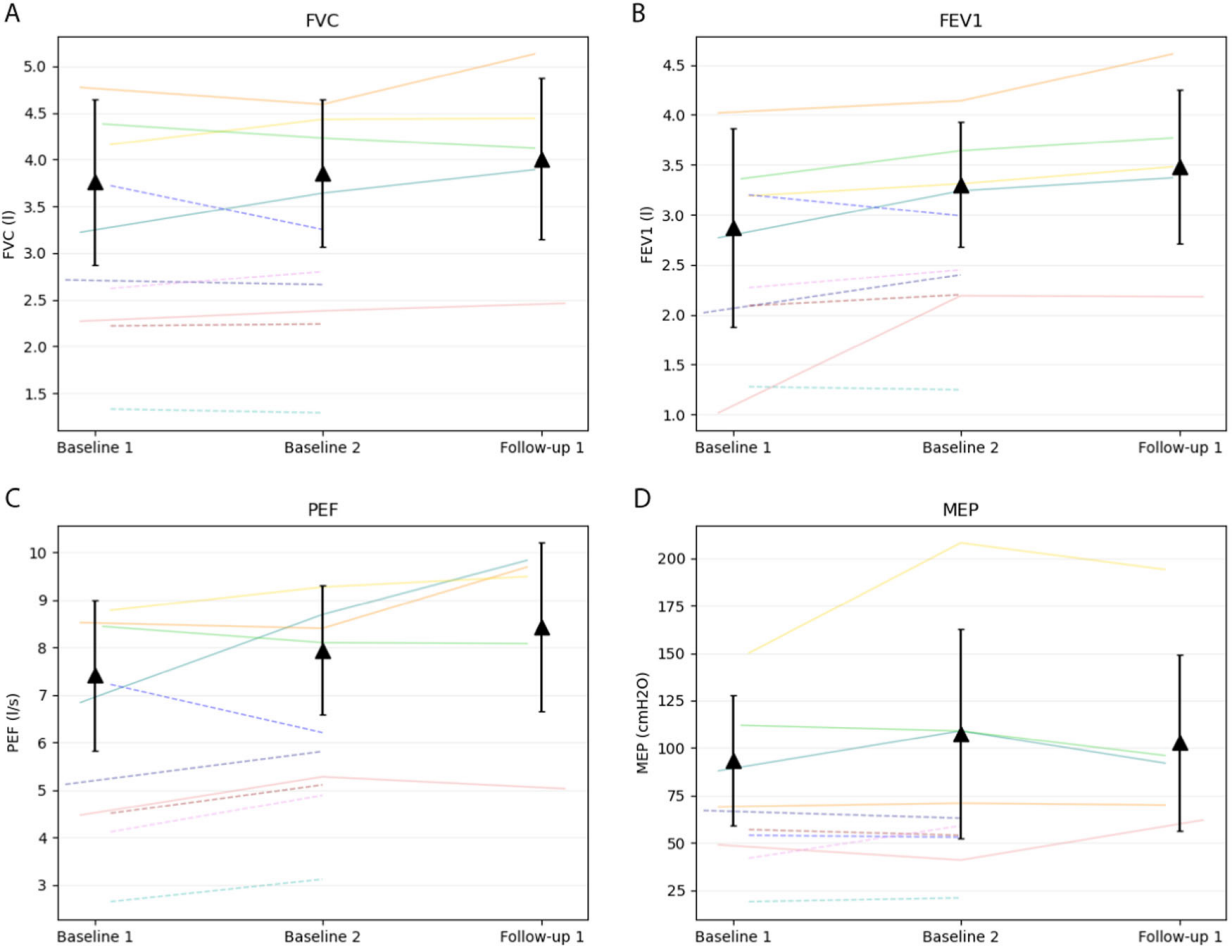
Change of expiratory outcomes. Graphical depiction of expiratory outcomes from Baseline 1 to Follow-up 1 of: (**A**) Forced Vital Capacity (FVC), (**B**) Force Expiratory Volume in 1 Second (FEV_1_), (**C**) Peak Expiratory Flow (PEF), and (**D**) Maximal Expiratory Pressure (MEP). Each color represents data from a single participant with lines showing change between assessment values. Solid lines show the data that were used in the means calculations while dotted lines represent individuals with missing data from Follow-up 1. The mean and 95% confidence intervals are plotted for each assessment day as black triangles. Participant 6 is represented by the color green.

**Fig. 6:**
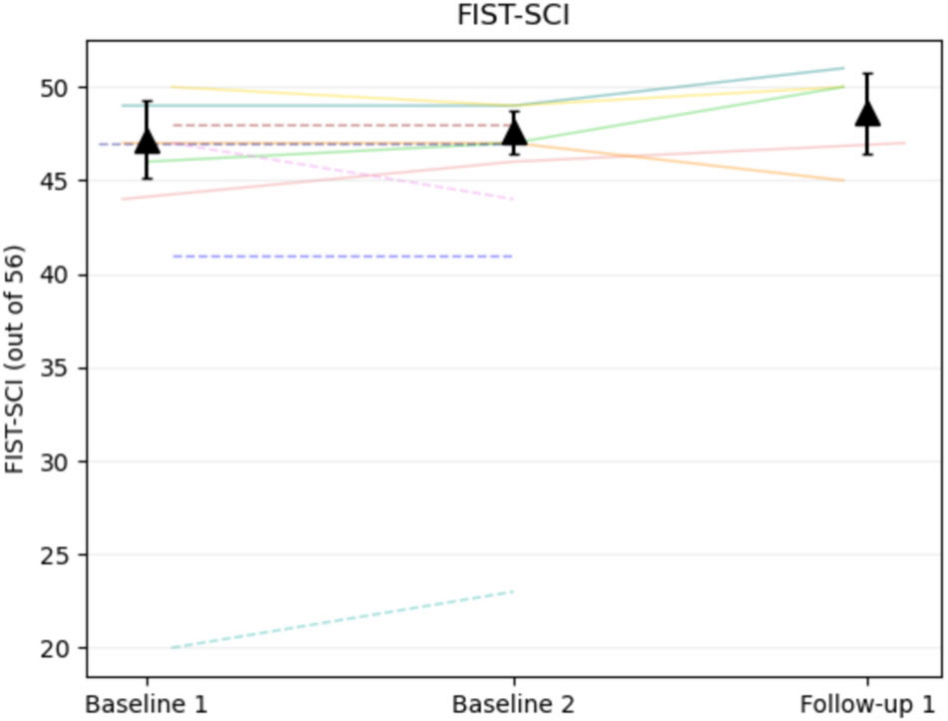
Change of functional seated balance. Graphical depiction of Function in Sitting Test for people with spinal cord injury (FIST-SCI) outcomes from Baseline 1 to Follow-up 1. Each color represents data from a single participant with lines showing change between assessment values. Solid lines show the data that were used in the means calculations while dotted lines represent individuals with missing data from Follow-up 1. The mean and 95% confidence intervals are plotted for each assessment day as black triangles. Participant 6 is represented by the color green.

The changes in all outcomes from BL1 to F1 can be found in Table 2 and in Figs. 4–6. Repeated measures ANOVA found significant effects for MIP (F(1.3,11.4)= 12.3, *p* = 0.003) and SMIP (F(2, 18) = 8.2, *p* = 0.003) between BL1, BL2, and F1, but not ID (*p* = 0.071). Mean MIP was significantly higher at F1 (mean: 148.3 cmH2O, 95% Confidence Interval: [116.9, 179.7]) than BL1 (121.4 cmH2O [99.7, 143.1], *p* = 0.030) and BL2 (116.7 cmH2O [93.9, 139.5], *p* = 0.009). Mean SMIP at F1 (740.9 PTU [549.6, 932.2]) was found to be significantly higher than BL2 (601.9 PTU [417.7, 786.1], *p* < 0.001) but not BL1 (718.0 PTU [520.4, 915.6], *p* = 1.0). Additionally, mean TP at F1 (22,836.0 PTU [18,151.4, 27,520.6]) was significantly greater than BL2 (17,995.3 PTU, [12,413.8, 23,576.8]) (t(9)= 2.880, *p* = 0.018). The largest observable percentage increase from BL2 to F1 occurred in TP, followed by SMIP and MIP (37%, 29%, and 28%, respectively).

**Table 2:** Respiratory and balance outcomes from baseline 1 to follow-up 1.

		TP	SMIP	MIP	ID	FVC	FEV1	PEF	MEP	FIST-SCI
BL1	*n*		10	10	10	5	5	5	5	5
	mean		718*	121.4*	14.4	3.8	2.9	7.4	93.6	47.2
	95% CI		[520.4, 915.6]	[99.7, 143.1]	[11.5, 17.3]	[2.5, 5.0]	[1.5, 4.3]	[5.2, 9.7]	[44.9, 142.3]	[44.2, 50.16]
	*POP*		91.6%	154.8%		105.8%	123.1%	129.2%	113.5%	
BL2	*n*	10	10	10	10	5	5	5	5	5
	mean	17995.3	601.9*	116.7*	12.7	3.9	3.3	8.0	107.6	47.6
	95% CI	[12413.8, 23576.8]	[417.7, 786.1]	[93.9, 139.5]	[10.5, 15.0]	[2.7, 5.0]	[2.4, 4.2]	[6.0, 9.9]	[29.4, 185.8]	[45.9, 49.3]
	*POP*		77.6%	148.8%		108.0%	145.1%	137.7%	136.7%	
F1	*n*	10	10	10	10	5	5	5	5	5
	mean	22836.0^	740.9*^	148.3*#	14.5	4.0	3.5	8.4	102.8	48.6
	95% CI	[18151.4, 27520.6]	[549.6, 932.2]	[117.0, 179.7]	[11.9, 17.2]	[2.8, 5.2]	[2.4, 4.6]	[5.9, 10.9]	[37.0, 168.6]	[45.5,51.7]
	*POP*		96.2%	187.7%		111.9%	152.2%	144.0%	121.9%	

Means, 95% confidence interval (CI), and percent of predicted values at each time point with available participants denoted (n). SMIP Percent Predicted values (Cahalin), MIP and MEP (Raab), and FVC, FEV1, and PEF (Mueller). *Main effect in group (*p* < 0 .05). ^#^Follow-up 1 is significantly different than Baseline 1 and Baseline 2. ^^^Follow-up 1 is significantly different than Baseline 2. Percent of predicted MIP and MEP values are based on Raab equations [48] [MIP = 62 + (lesion specific constant) + 18(if male) + 0.24(kg); MEP = 72 + (lesion specific constant) + 18 (if male)]. Percent of predicted FVC, FEV1, PEF values are based on Mueller equations [49] [FVC = −1.219 + (lesion specific constant) + .645(if male) − 0.026(age) +0.024(cm) + 0.010 (kg); FEV1 = −0.798 + (lesion specific constant) + 0.505(if male) - 0.025(age) + 0.021(cm) + 0.006 (kg); PEF = –1.327 + (lesion specific constant) + 1.049(if male) - 0.031(age) + 0.032(cm) + 0.015 (kg)]. Percent of predicted SMIP values are based on Cahalin equations [47] [SMIP(male) = 12.6(cm) – 9.95(age) – 1054.1 or SMIP = 630.9 – 4.19(age)]. Weight in kilograms (kg), Height in centimeters (cm), Level of injury (LOI), spinal cord injury (SCI). BL1: Baseline 1, BL2: Baseline 2, F1: Follow-up 1, F2: Follow-up, F3: Follow-up 3, F4: Follow-up 4.

*POP* percent of predicted, *TP* total power, *SMIP* sustained maximal inspiratory pressure, *MIP* maximal inspiratory pressure, *ID* inspiratory duration, *FVC* forced vital capacity, *FEV1* forced expiratory volume in 1 Second, *PEF* peak expiratory flow, *MEP* maximal expiratory pressure, *FIST-SCI* function in sitting test for individuals with spinal cord injury.

**Fig. 7:**
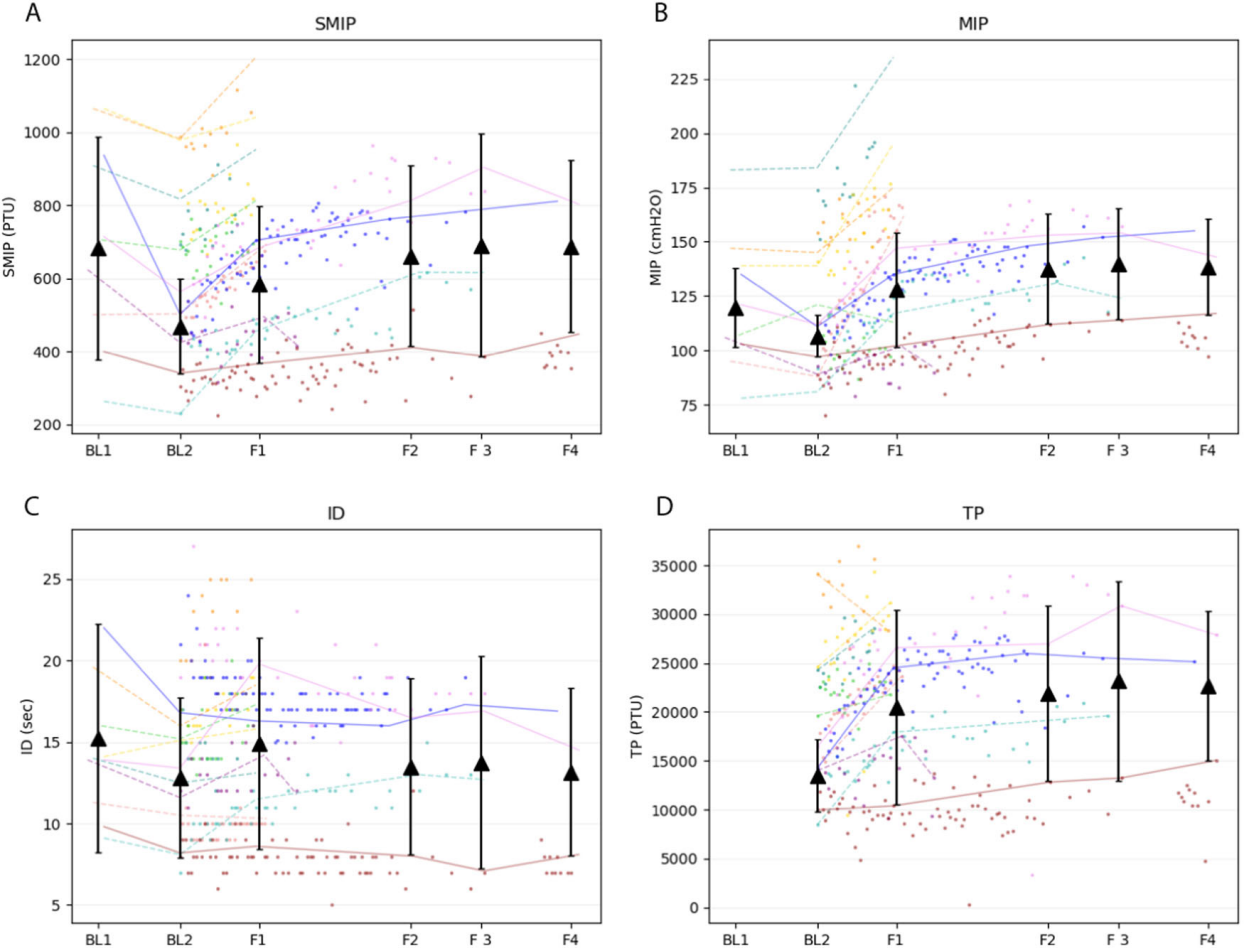
Change of inspiratory outcomes (*n* = 3). Graphical depiction of inspiratory outcomes from Baseline 1 to follow-up 4 of: (**A**) sustained Maximal Inspiratory Pressure (SMIP), (**B**) maximal Inspiratory Pressure (MIP), (**C**) inspiratory duration (ID), and (**D**) total power (TP). Each color represents data from a single participant with lines showing change between assessment values. Solid lines show the data that were used in the means calculations while dotted lines represent individuals who did not complete all phases and follow-up assessments of the study. Dots represent the highest value of the variable achieved per session. The mean and 95% confidence intervals are plotted for each assessment day as black triangles. Participant 6 is represented by the color green. BL1 baseline 1, BL2 baseline 2, F1 follow-up 1, F2 follow-up 2, F3 follow-up 3, F4 follow-up 4, PTU pressure time units.

Three participants completed all phases of the study. Inspiratory data from the three participants who completed all phases and assessments can be found in Table 3 and Fig. 7. The highest recorded TP, SMIP, and MIP scores were observed at F3 for these individuals which related to an increase from BL2 of 71%, 47%, and 31%, respectively. The highest recorded ID was found at F1.

**Table 3:** Inspiratory performance of the 3 participants who completed all phases of the study.

		TP	SMIP	MIP	ID
BL1	mean		683.3	119.7	15.2
	95% CI		[988.1, 378.6]	[101.5, 137.8]	[8.2, 22.3]
	POP		93.2%	171.4%	
BL2	mean	13536.7	468.7	106.7	12.8
	95% CI	[9817.1, 17256,2]	[338.9, 598.5]	[97.2, 116.2]	[7.9, 17.7]
	POP		66.3%	154.6%	
F1	mean	20495.0	584.7	128.0	14.9
	95% CI	[10538.2, 30451.8]	[370.1, 799.3]	[101.6, 154.4]	[8.4, 21.4]
	POP		80.9%	181.9%	
F2	mean	21927.3	662.0	137.7	13.5
	95% CI	[12995.9, 30858.7]	[414.3, 909.7]	[112.4, 163.0]	[8.1, 18.9]
	POP		91.7%	196.0%	
F3	mean	23204.0	691.3	140.0	13.8
	95% CI	[12995.9, 33412.1]	[385.6, 997.1]	[114.5, 165.5]	[7.2, 20.3]
	POP		94.7%	214.9%	
F4	mean	22687.3	687.3	138.3	13.2
	95% CI	[15035.4, 30339.2]	[452.7, 921.9]	[116.4, 160.3]	[8.0, 18.3]
	POP		95.6%	197.7%	

Means, 95% Confidence Interval (CI), and percent of predicted values at each time point. Percent of predicted MIP values are based on Raab equations [48] [MIP = 62 + (lesion specific constant) + 18(if male) + 0.24(kg)]. Percent of predicted SMIP values are based on Cahalin equations [47] [SMIP(male) = 12.6(cm) – 9.95(age) – 1054.1 or SMIP = 630.9–4.19(age)].

*BL1* baseline 1, *BL2* baseline 2, *F1* follow-up 1, *F2* follow-up, *F3* follow-up 3, *F4* follow-up 4, *POP* percent of predicted, *TP* total power in pressure time units, *SMIP* sustained maximal inspiratory pressure in pressure time units, *MIP* maximal inspiratory pressure in cmH2O, *ID* inspiratory duration in seconds.

## Discussion

The primary objective of this study was to assess the ability of individuals with chronic SCI to complete daily, high-intensity IMT sessions in their homes. This is the first IMT clinical trial in SCI to report adherence based on virtually collected data from a cloud-based interface, which, may decrease participant burden in maintaining a training log. Participants completed 69.3% of the recommended training sessions (~5 days/week) in the first month which resulted in significant improvements in MIP, SMIP, and TP at F1. The improvements appear to be maintained in individuals who completed all phases of the study.

Average adherence data from the first month of the current study was consistent with findings from additional phases (65.6%) suggesting that long-term and short-term IMT adherence rates are similar. These rates are lower than the compliance rate of 98% reported by Gee et al. in a six week RMT program performed by six wheelchair rugby athletes who trained five days per week at an intensity of up to 80% of their MIP and MEP [31]. Though the compliance rates differed, both participant groups trained about five days/week [31]. While adherence rates were maintained above 65% in IMT Phases 1-3, only one of three participants trained during IMT Phase 4. The decrease in participation during IMT Phase 4 is similar to prior reports [15] and highlights the importance of weekly monitoring. Overall, we found that a high-intensity IMT home exercise program with weekly monitoring results in good adherence for individuals with SCI.

High intensity, high frequency IMT may not be appropriate for all individuals with SCI based on the data from P6, who had tetraplegia and an extensive history of smoking from a young age. It is possible that the decrease in RF noted prior to limiting training frequency was due to overtraining. The concept of respiratory overtraining has been discussed in previous IMT studies where performance of some RF variables decreased throughout a training program [50, 51]. Thus, the high intensity and frequency of IMT proposed in this study may have produced an overtraining effect in P6, highlighting the need for future investigation of IMT dose in patients with SCI and possible smoking-related lung dysfunction.

The results of repeated measures ANOVA showed a significant improvement in mean SMIP and MIP over the first month of training even with data from P6 included. The 28% increase of mean MIP from BL2 was very similar to the 32% mean increase in MIP reported in a sham-controlled clinical trial in which participants with SCI completed six weeks of resistive RMT [15]. Mean TP significantly increased from BL2 to F1, which likely represents an improvement in respiratory power and endurance as participants created more forceful breaths throughout a training session. The significant TP improvement from BL2 to F1 (37%) may be representative of musculature hypertrophy. Evidence of rapid muscle adaptation was reported by DeFritas et al., who found significant increases in skeletal thigh muscle cross sectional area at three weeks, and in maximal volitional contraction at four weeks in neurologically intact individuals completing resistance training at 80% of a one-repetition maximal effort [52]. The respiratory muscles that were fully innervated in participants in the current study may have responded similarly to this high-intensity training.

There was no significant difference in ID between BL1 and F1 in the total sample. Individuals with stronger respiratory muscles may reach TLC faster than those with weak musculature, evidenced by the report of a significant negative relationship between ID and LOI [4]. This relationship could help to explain why there appears to be a decrease in ID while MIP, SMIP, and TP were maintained or improved in the three individuals who completed all study phases. The plateau or decrease in time to reach total lung capacity paired with the increase in MIP and SMIP outcomes may be beneficial and representative of improved phasic strength of the respiratory musculature and has not been previously reported as a training effect of IMT in people with SCI.

While ID appears to decrease, MIP, SMIP and TP appear to be maintained or improve, even when participants trained once a week. The statistical significance of changes after F1 could not be determined but the means of MIP, SMIP, and TP for the three individuals who completed all phases of the study did not fall below F1 levels, even when training frequencies were reduced during IMT Phase 3 and 4. The participant shown in red was the only participant to train during IMT Phase 4 (unmonitored, self-selected IMT frequency) and experienced improvements from F3 to F4. Romer and McConnell reported that neurologically intact individuals who had been performing IMT six times per week for nine weeks and then dropped to two times per week maintained the improvements gained over the first nine weeks of training [53]. However, inspiratory performance decreased significantly in a separate group who performed the same initial nine-week training but had no maintenance IMT program [53]. The results of Romer and McConnell resemble the changes due to both maintenance efforts and detraining reported in IMT Phase 3 and 4 of this study.

The impact of unsupervised IMT on expiratory measures was not able to be determined due to the limited data collected. Meta-analyses in patients with SCI have found RMT to have a positive effect on FVC [54], VC [13, 54, 55], MIP [13, 54, 55], MEP [13, 54, 55], inspiratory capacity [13], and maximal voluntary ventilation [54], but not FEV1 [13, 54, 55]. However, the training programs in these meta-analyses included supervised IMT, EMT, RMT, singing, normocapnic hyperpnea, and isocapnic hyperpnea making it difficult to determine the reported effects being due to a specific type of training. It is still unclear the impact, if any, of unsupervised IMT on expiratory assessments.

The significant inspiratory changes reported in this study may be more relevant than the lack of PF results. Boswell-Ruys et al. found that the MEP, FVC, FEV1, and PEF during a cough of individuals with SCI who performed RMT was not significantly different from the sham group even though MIP differed substantially between groups [15]. Importantly, individuals with a significant improvement in MIP were less likely to be hospitalized for RC over the next year, providing evidence that inspiratory performance may be more indicative of pulmonary health than expiratory assessments [15]. Thus, the significant inspiratory performance findings in this study may be more clinically important than possible PF results.

Last, the data from BL1 and BL2 examining balance enforce the stability of the FIST-SCI as there was just a .7% change in the mean score of the cohort during the control period. The increase of 1 point in the FIST-SCI between BL2 and F1 is less than the FIST-SCI MDC of four [41]. Four of the five individuals included in the means calculations for the FIST-SCI had paraplegia and all five individuals scored over 45, indicating a high likelihood of the ability to transfer independently [41]. People with paraplegia have better balance and RF than individuals with cervical SCI, and the relationship between SMIP and FSB is stronger in individuals with tetraplegia [23, 45, 56, 57]. IMT may have a greater effect on individuals with poorer baseline balance and RF than shown in this cohort. These data could be used to power a future trial.

### Limitations

The data in the current study were limited by the COVID-19 pandemic. The observed power calculated for the repeated measures ANOVA analyses was greater than 0.80. It is difficult to interpret the significant change in SMIP from BL1 to BL2. The three individuals who participated in all phases of the study accounted for the highest negative percent change in the control period. BL2 testing for these three participants occurred in the first two weeks of March 2020. It cannot be ruled out that COVID-related anxiety or undiagnosed infection impacted RF, specifically SMIP. SMIP has been identified as the inspiratory assessment most related to overall respiratory function, and may be sensitive to subtle changes in health state [4].

Overall, the compliance percentage and RF improvements found in this study support the use of high-intensity IMT home exercise programs for the chronic SCI population. It is highly recommended that home IMT protocols be monitored intermittently to preserve compliance and safety. Future research should further explore the effect of IMT on balance and PF in the SCI population

## Supplementary information

Appendix 1

## Data Availability

Individual data are represented in the figures of this paper. Additional data are available from the corresponding author on reasonable request.
