# Supplementary material for: Adherence to and impact of home-based high-intensity IMT in people with spinal cord injury: a pilot study
Source: Spinal Cord Ser Cases. 2022 Oct 30;8:85. doi: 10.1038/s41394-022-00551-5 (PMC9617741; doi:10.1038/s41394-022-00551-5)
Supplement: Supplementary file 1 — Appendix 1 [file 41394_2022_551_MOESM1_ESM.docx]

Appendix 1

A repeated measures design was planned to assess inspiratory muscle performance, PF, and FSB at multiple time points. The original study plan included a wash in control period of four weeks where participants were asked to not greatly alter their daily routines, followed by a training period of four weeks. During the training period participants were instructed to use the PrO2FIT device daily for 4 weeks and record training information in their training log. The original study concluded with an efficacy period where individuals stopped training and returned to their regular daily activities. Planned testing days included study baseline (BL1), prior to initiation of the training period (BL2), at the end of the training period prior to the efficacy period (F1), and at the completion of the efficacy period (F2) where all outcomes including MIP, SMIP, ID, MEP, FVC, FEV1, PEF and FIST-SCI, would be assessed. TP would have only been assessed at BL2 and F1. However, the COVID-19 pandemic impacted the study course. Due to safety concerns all recruitment and in person assessment was stopped on March 13, 2020. Prior to March 13, 21 individuals had consented to participate in the study, 18 people completed BL1, 12 of those participants completed BL2, 6 of those subjects completed F1, and 6 individuals were in the training period. After study modifications, 5 of the 6 participants in the training period (IMT Phase 1) agreed to continue to participate in a virtual extension of the study outlined in the full manuscript.

Further, the initial study planned to assess autonomic activity, but the limited data collected prevented analysis. Individuals with pacemakers or individuals being treated with beta-blockers were excluded from the study as those interventions would limit the utility of the autonomic assessments.
